# Long‐term mortality associated with depression among South Korean survivors of extracorporeal membrane oxygenation

**DOI:** 10.1002/brb3.2218

**Published:** 2021-05-30

**Authors:** Hye Youn Park, Hyoung‐Won Cho, In‐Ae Song, Sukyoon Lee, Tak Kyu Oh

**Affiliations:** ^1^ Department of Psychiatry Seoul National University Bundang Hospital Seongnam Korea; ^2^ Department of Cardiology Seoul National University Bundang Hospital Seongnam South Korea; ^3^ Department of Anesthesiology and Pain Medicine Seoul National University Bundang Hospital Seongnam South Korea; ^4^ Department of Neurology Inje University College of Medicine Busan Republic of Korea

**Keywords:** depression, epidemiology, extracorporeal membrane oxygenation, intensive care units

## Abstract

**Introduction:**

Depression is an important sequela in critically ill patients. However, its prevalence after extracorporeal membrane oxygenation (ECMO) therapy and its association with long‐term mortality remain controversial.

**Methods:**

Data were extracted from the South Korean National Health Insurance Service database in this population‐based cohort study. Adults who received ECMO therapy from 2006 to 2014 were included. Survivors of ECMO were defined as patients who underwent ECMO and survived over 365 days after the initiation of ECMO therapy.

**Results:**

A total of 3,055 survivors of ECMO were included in the final analysis. They were classified into the pre‐ECMO depression group (*n* = 275 [9.0%]), post‐ECMO depression group (*n* = 331 [10.8%]), and other ECMO survivor group. In the multivariable Cox regression model, a 1.52‐fold higher mortality was observed in the post‐ECMO depression group than in the other groups (hazard ratio, 1.52; 95% confidence interval, 1.17–1.96; *p* = .002). However, there was no statistically significant difference between the pre‐ECMO depression group and the other groups (*p* = .075).

**Conclusions:**

The prevalence of pre‐ and post‐ECMO depression was 9.0% and 10.8%, respectively. Additionally, post‐ECMO depression was associated with an increased 5 year all‐cause mortality; however, pre‐ECMO depression was not.

## Graphical abstract

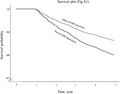

## 1 INTRODUCTION

Extracorporeal membrane oxygenation (ECMO) is an artificial extracorporeal support for cardiopulmonary failure refractory to conventional therapies (Combes et al., 2018; Gattinoni et al., 2011). Its clinical indications include heart failure, intractable arrhythmia, heart inflammation, pulmonary hypertension, trauma, postcardiac surgery, respiratory failure, and acute respiratory distress syndrome (ARDS) (Aneman et al., 2018; Brodie et al., 2019; Eckman et al., 2019; Lafc et al., 2014; Tramm et al., 2015). According to the extracorporeal life support organization registry report, the incidence of ECMO therapy has been on the rise, globally, from 2006 to 2017 (Nasr et al., 2019). In South Korea, the incidence of ECMO therapy increased by 2.5‐fold between 2009 and 2014 (Tay et al., 2019). In 2020, ECMO was used worldwide as a rescue therapy in many critically ill patients with coronavirus disease 2019 (Barbaro et al., 2020; Yang et al., 2020). Thus, the clinical utility of ECMO will be more emphasized in the future.

As approximately 40%–60% of patients treated with ECMO survive to hospital discharge (Nasr et al., 2019), over 40% of patients undergoing ECMO may be expected to survive. A previous study reported that the 87% of ECMO patients who survived the first 90 days after ECMO therapy survived for 5 years (von Bahr et al., 2017), suggesting that quality of life among survivors of ECMO is an important public health issue. Depression may be an important psychological sequelae of ECMO therapy. It is commonly observed in survivors of critical illness who were admitted to the intensive care unit (ICU) (Battle et al., 2015), with a reported prevalence of 46% in a single‐center retrospective study. (Battle et al., 2015) The prevalence of depression was 40% in survivors of critical illness from 26 ICUs in the United Kingdom (Hatch et al., 2018). Recently, single‐center cohort studies have reported that the prevalence of depressive symptoms among survivors of ECMO ranged from 15%–42% (O'Brien et al., 2020; Sanfilippo et al., 2019; Sylvestre et al., 2019). However, these studies had small sample sizes (O'Brien et al., 2020; Sanfilippo et al., 2019; Sylvestre et al., 2019) and the association of post‐ECMO depression with long‐term mortality was not identified. Recently, the long‐term quality of life following ECMO therapy has been emphasized as an important health issue (Wilcox et al., 2020), and the early identification and treatment of mental illnesses, such as depression, following ECMO therapy among survivors might improve long‐term outcomes regarding quality of life (Knudson et al., 2019). Furthermore, depression following critical illness was associated with an increased relative long‐term mortality among survivors of critical illness (Hatch et al., 2018). Therefore, depression following ECMO therapy might be associated with increased long‐term mortality among survivors of ECMO; however, there have been no studies on this topic.

Therefore, we aimed to investigate the prevalence of pre‐ and post‐ECMO depression and examine its impact on the long‐term mortality of survivors of ECMO, using data from the National Health Insurance Service (NHIS) database in South Korea.

## 2 MATERIAL AND METHODS

### 2.1 Study design and approval

This population‐based cohort study was conducted according to the Strengthening the Reporting of Observational Studies in Epidemiology guidelines (von Elm et al., 2014). The study protocol was approved by the Institutional Review Board of Seoul National University Bundang Hospital (X‐2001–586–902) and the Health Insurance Review and Assessment Service (NHIS‐2020–1–125). The requirement for informed consent was waived because the data analyses were performed retrospectively using anonymized data derived from the South Korean NHIS database.

### 2.2 NHIS **database and study population**

This study used data from the health records obtained from the South Korean NHIS database. In South Korea, information concerning disease diagnoses and prescription of drugs and/or procedures is registered in the NHIS database so that patients can receive financial support for treatment. Data were extracted by an unaffiliated, independent medical record technician at the NHIS center. All adult patients (age ≥18 years) who received ECMO therapy between 2006 and 2014 were included. Those who only received ECMO therapy during surgery were excluded. The prescription codes for ECMO in the database were O1901–1904, and the use of a Novalung (O1905) was not considered as ECMO in this study. We defined survivors of ECMO as those who had survived over 365 days after the initiation of ECMO therapy.

### 2.3 Depression

The diagnosis of depression was evaluated using the following International Classification of Diseases, 10th revision (ICD‐10) codes in the NHIS database: F20.4, F31.3–F31.5, F32*, F33*, F34.1, F41.2, and F43.2. The survivors of ECMO were classified as follows: (1) pre‐ECMO depression group (diagnosed with depression before ECMO therapy), (2) post‐ECMO depression group (no history of depression but newly diagnosed with depression within 1 year after ECMO therapy), and (3) other ECMO survivors (not diagnosed with depression before or after ECMO therapy). In South Korea, patients with depression only receive compensation for their medical expenses if they are registered using ICD‐10 codes in the NHIS database by a physician or psychiatrist. Therefore, the NHIS database includes almost all patients with depression if they had any treatment for depression.

### 2.4 Study outcomes

The primary endpoint of this study was the 5 year all‐cause mortality among survivors of ECMO, which was defined as death within 5 years of undergoing ECMO therapy. The patients’ dates of death were extracted until 1 April 2020, and survival times were calculated from the date that ECMO therapy was started to the date of death or 1 April 2020, whichever occurred first. The secondary endpoint of this study was the development of post‐ECMO depression, which was evaluated within 365 days from the date of starting ECMO therapy.

### 2.5 Measurements of confounders

The data on confounders were extracted using ICD‐10 codes registered within 1 year of starting the ECMO therapy and included demographic characteristics (age, sex, and place of residence at the time of ECMO therapy [Seoul, metropolitan city, and other areas]); annual income; underlying disability; other underlying psychiatric illness, such as post‐traumatic stress disorder (PTSD, ICD‐10: F43.1) and anxiety disorder (F41); and Charlson comorbidity index (CCI). The CCI was used to reflect the comorbid status of ECMO survivors at ECMO therapy because the CCI can predict in‐hospital mortality among hospitalized patients (Sundararajan et al., 2004). The CCI was calculated as the sum of the 17 individual underlying diseases listed in Table S1 (1 point for 10 diseases, 2 points for 4 diseases, 3 points for 1 disease, and 6 points for 2 diseases); therefore, the range of CCI was from 0 (best score) to 27 (worst score) in this study. In addition, information on the other treatments administered, length of hospital stay, duration of ECMO therapy, annual case volume of ECMO therapy, and main diagnosis at the time of ECMO therapy were collected. The annual case volume of ECMO therapy was calculated as the total ECMO cases/9 years to reflect the capability of the ECMO centers (Barbaro et al., 2015), and it was divided into four groups (Q1 <14, Q2: 15–29, Q3: 30–64, and Q4 > 64). In South Korea, all individuals with disabilities are registered in the NHIS database to receive various benefits from the social welfare system. Disabilities were divided into 15 types: physical disabilities, brain lesion disabilities, visual disturbances, hearing disturbance, speech disabilities, autism, intellectual disorder, mental disorder, renal disorder, heart diseases, respiratory disorders, hepatopathy, intestinal fistulae, urinary fistulae, and epilepsy. Additionally, each disability was assigned one of six grades based on severity; we grouped the severity grades into two (1–3, severe disability; and 4–6, mild to moderate disability). The main diagnoses at the time of ECMO therapy were classified into four groups: (1) potential venoarterial (VA) ECMO group (cardiovascular disease or shock), (2) potential venovenous (VV) ECMO group (ARDS or respiratory failure), (3) potential extracorporeal cardiopulmonary resuscitation (ECPR) group (post‐cardiac arrest), and (4) others.

### 2.6 Statistical analysis

The clinicoepidemiological characteristics of the survivors of ECMO are presented as means with standard deviations for continuous variables and numbers with percentages for categorical variables. First, we fit the multivariable logistic regression model for development of post‐ECMO depression within 365 days following ECMO therapy among survivors. All covariates were included in the multivariable logistic regression model for adjustment, except for pre‐ECMO depression, which was not included because the pre‐ECMO group had been treated for depression both before and after ECMO therapy.

Next, we fit the multivariable Cox regression model for the 5 year all‐cause mortality among the survivors of ECMO to investigate whether pre‐ and post‐ECMO depression were associated with 5 year all‐cause mortality. All covariates were included in the model for adjustment. Additionally, we performed sensitivity analyses according to pre‐ and post‐ECMO depression with or without other psychiatric illness (PTSD or anxiety disorder) using multivariable Cox regression modeling. Finally, we performed the subgroup analyses according to main diagnosis at ECMO therapy to examine the impact of potential type of ECMO and ECPR on associations of pre‐ and post‐ECMO depression with 5 year all‐cause mortality among survivors. We confirmed that there was no multicollinearity in all the multivariable models involving the entire cohort, with a variance inflation factor of <2.0. The results of the Cox regression are presented as hazard ratios (HRs) with 95% confidence intervals (CIs), and log‐log plots were used to confirm that the central assumption of multivariable Cox model was satisfied. The results of the logistic regression models are presented as odds ratios (ORs) with 95% CIs. The Hosmer–Lemeshow test was used to confirm the goodness of fit of the multivariable models at *p* > .05. R software (version 4.0.3; R Foundation for Statistical Computing) was used for all analyses, and a *p*‐value < .05 was considered statistically significant.

## 3 RESULTS

**FIGURE 1:**
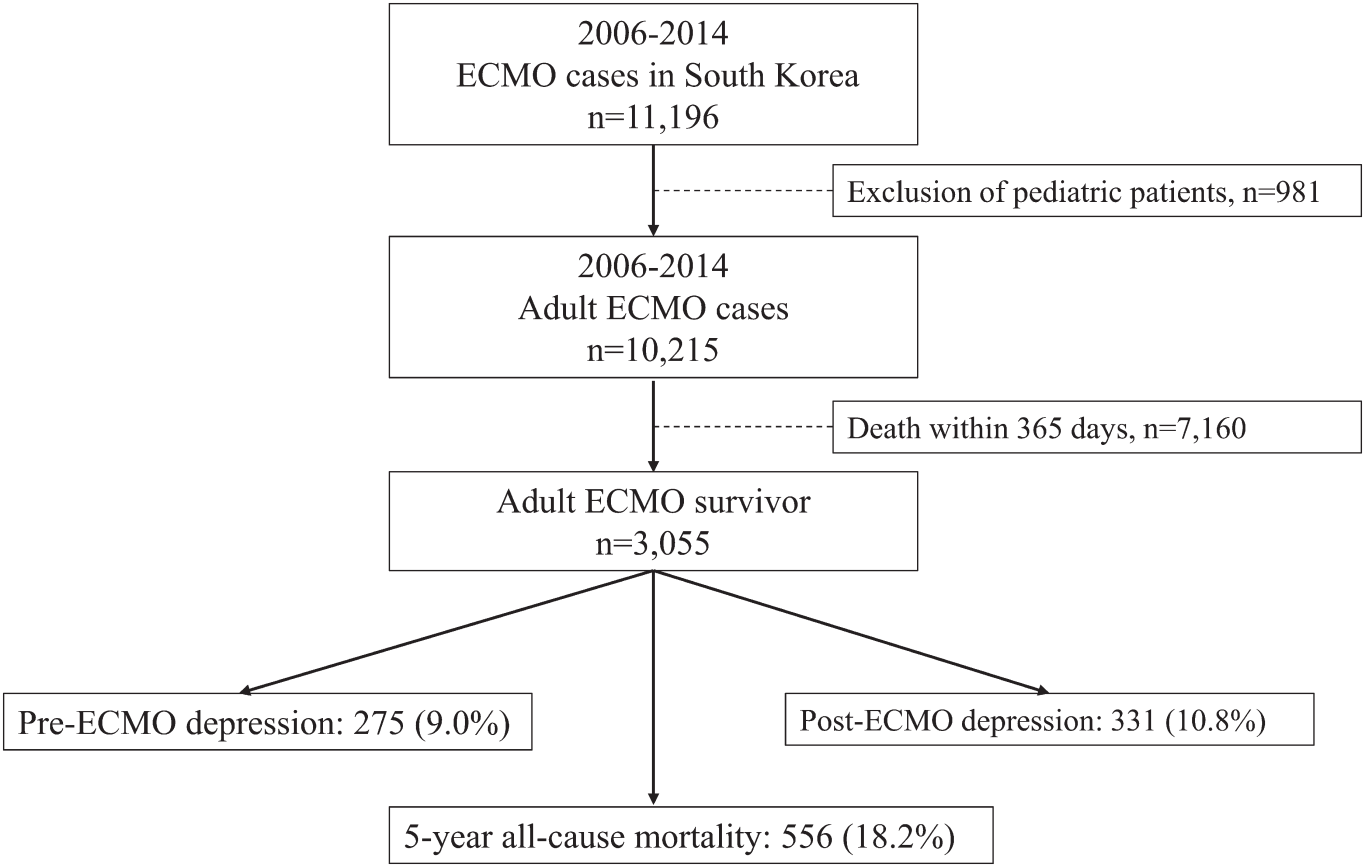
Flowchart depicting the patient. selection process ECMO, extracorporeal membrane oxygenation

From 2006 to 2014, 11,196 patients received ECMO therapy at 72 hospitals in South Korea. After excluding 981 patients aged <18 years, 10,215 patients were screened. Of these patients, 3,055 (29.9%) survived for more than 365 days after the initiation of ECMO therapy and were included in this study. Among them, 275 (9.0%) and 331 (10.8%) were assigned to the pre‐ and post‐ECMO depression groups, respectively. Five‐year all‐cause mortality occurred in 556 (18.2%) survivors of ECMO therapy. Figure 1 presents a flowchart depicting patient selection. The clinicoepidemiological characteristics of the patients are presented in Table 1.

**TABLE 1:** The clinicoepidemiologic characteristics of ECMO survivors from 2006 to 2014 (*n* = 3,055)

Variable	Number (%)	Mean (*SD*)
Age, years		53.4 (15.2)
Sex, male	2,032 (66.5)	
Residence at ECMO therapy		
Capital city (Seoul)	770 (25.2)	
Other metropolitan city	631 (20.7)	
Other area	1,654 (54.1)	
Year of ECMO therapy		
2006	114 (3.7)	
2007	144 (4.7)	
2008	186 (6.1)	
2009	223 (7.3)	
2010	271 (8.9)	
2011	342 (11.2)	
2012	439 (14.4)	
2013	580 (19.0)	
2014	756 (24.7)	
Annual income level at ECMO therapy		
Q1 (Lowest)	748 (24.5)	
Q2	535 (17.5)	
Q3	763 (25.0)	
Q4 (Highest)	1,009 (33.0)	
Annual Case volume of ECMO therapy		
Q1 <14	508 (16.6)	
Q2: 15–29	735 (24.1)	
Q3: 30–64	100 (34.4)	
Q4 >64	762 (24.9)	
Charlson comorbidity index		2.8 (2.2)
Pre‐ECMO depression	275 (9.0)	
Post‐ECMO depression	331 (10.8)	
Underlying PTSD	96 (3.1)	
Underlying anxiety disorder	819 (26.8)	
Underlying disability		
Mild to moderate	464 (15.2)	
Severe	340 (11.1)	
Length of hospital stay, days		24.6 (15.2)
Duration of ECMO therapy, days		6.2 (10.3)
5 year all‐cause mortality	556 (18.2)	
Main diagnosis at ECMO therapy		
Cardiovascular disease or Shock	1,787 (58.5)	
ARDS or respiratory failure	281 (9.2)	
Postcardiac arrest	135 (4.4)	
Others	852 (27.9)	

Note

Others: cancer, liver failure, sepsis, trauma, injury and burn, post‐procedures, connective tissue disease, GI tract disease, cerebrovascular disease, urinary tract disease, nervous system disease, endocrine disease, musculoskeletal disease, event at pregnancy or childbirth, poisoning or drug intoxication, hematologic disorder, congenital malformation, unknown.

Abbreviations: ARDS, acute respiratory distress syndrome; ECMO, Extracorporeal membrane oxygenation; GI, gastrointestinalPTSD, post‐traumatic disorder; *SD*, standard deviation.

### 3.1 Factors associated with post‐ECMO depression

Table 2 shows the results of the multivariable logistic regression model for the development of depression within 1 year after ECMO therapy. An increase in age by 1 year was associated with a 1% increase in the development of post‐ECMO depression (OR, 1.01; 95% CI, 1.00–1.02; *p* = .032), and an increase in the length of hospital stay by 1 day was associated with a 2% increase in the development of post‐ECMO depression. Furthermore, the ECMO survivors with PTSD and anxiety disorder showed a threefold (OR, 3.00; 95% CI, 1.83–4.90; *p* < .001) and 1.37‐fold (OR, 1.37; 95% CI, 1.06–1.77; *p* = .016) increased risk for the development of post‐ECMO depression, respectively.

**TABLE 2:** Multivariable logistic regression model for development of post‐depression within 365 days following ECMO therapy among survivors

Variable	Multivariable model	*p*‐value
	OR (95% CI)	
Age, years	1.01 (1.00, 1.02)	.032
Sex, male	0.95 (0.74, 1.22)	.686
Residence at ECMO treatment		
Capital city (Seoul)	1	
Other metropolitan city	1.26 (0.89, 1.80)	.193
Other area	0.99 (0.73, 1.34)	.957
Year of ECMO treatment		
2006	1	
2007	3.99 (1.11, 14.33)	.034
2008	1.63 (0.42, 6.30)	.480
2009	3.35 (0.96, 11.71)	.058
2010	3.42 (1.00, 11.73)	.050
2011	2.86 (0.84, 9.71)	.093
2012	5.13 (1.56, 16.89)	.007
2013	5.15 (1.58, 16.78)	.007
2014	5.23 (1.61, 16.98)	.006
Annual income level at ECMO treatment		
Q1 (Lowest)	1	
Q2	0.61 (0.41, 0.92)	.017
Q3	0.99 (0.72, 1.37)	.959
Q4 (Highest)	0.94 (0.69, 1.28)	.698
Annual case volume of ECMO therapy		
Q1 <14	1	
Q2: 15–29	0.74 (0.53, 1.04)	.080
Q3: 30–64	0.71 (0.51, 0.99)	.044
Q4 >64	0.82 (0.57, 1.18)	.282
Charlson comorbidity index, points	1.03 (0.98, 1.09)	.284
Underlying PTSD	3.00 (1.83, 4.90)	<.001
Underlying anxiety disorder	1.37 (1.06, 1.77)	.016
Underlying disability		
Mild to moderate	1.01 (0.72, 1.41)	.955
Severe	0.98 (0.67, 1.43)	.897
Duration of ECMO therapy	1.01 (0.99, 1.02)	.351
Length of hospital stay, days	1.02 (1.01, 1.02)	<.001
Main diagnosis at ECMO therapy		
Cardiovascular disease or Shock	1	
ARDS or respiratory failure	1.14 (0.75, 1.74)	.538
Post‐cardiac arrest	1.39 (0.80, 2.41)	.249
Others	1.16 (0.86, 1.56)	.339

Note

Hosmer–Lemeshow: Chi‐square, 7.02, *df* = 8, *p* = .534. Others: cancer, liver failure, sepsis, trauma, injury and burn, post‐procedures, connective tissue disease, GI tract disease, cerebrovascular disease, urinary tract disease, nervous system disease, endocrine disease, musculoskeletal disease, event at pregnancy or childbirth, poisoning or drug intoxication, hematologic disorder, congenital malformation, unknown.

Abbreviations: ARDS, acute respiratory distress syndrome; CI, confidence interval; ECMO, Extracorporeal membrane oxygenation; GI, gastrointestinalOR, odds ratio; PTSD, post‐traumatic disorder.

### 3.2 Five‐year all‐cause mortality

Table 3 shows the results of the multivariable Cox regression analysis for the 5 year all‐cause mortality among the survivors of ECMO. A 1.52‐fold higher mortality was observed in the post‐ECMO depression group than in the other groups (HR, 1.52; 95% CI, 1.17–1.96; *p* = .002). The survival plot derived from the multivariable model showed a similar trend as that shown in Figure S1. However, there was no statistically significant difference for the 5 year all‐cause mortality between the pre‐ECMO depression group and the other groups (HR, 0.74; 95% CI, 0.63–1.13; *p* = .075). In addition, an increase in age by 1 year was associated with a 4% increase in the 5 year all‐cause mortality (HR, 1.04; 95% CI, 1.04–1.05; *p* < .001). ECMO survivors with underlying disability had a 1.94‐fold higher 5 year all‐cause mortality than ECMO survivors without disability (HR, 1.94; 95% CI, 1.55–2.43; *p* < .001). Compared to the ECMO survivor who received ECMO therapy for cardiovascular disease or shock, the ECMO survivors who received ECMO therapy for ARDS or respiratory failure and postcardiac arrest showed 1.48‐fold (HR, 1.48; 95% CI, 1.07–2.05; *p* = .018) and 1.99‐fold (HR, 1.99; 95% CI, 1.33–2.97; *p* = .001) higher 5 year all‐cause mortality, respectively.

**TABLE 3:** Multivariable Cox regression model for 5 year all‐cause mortality among ECMO survivors

Variable	Multivariable model	*p*‐value
	HR (95% CI)	
Pre‐ECMO depression (versus control group)	0.74 (0.63, 1.13)	.075
Post‐ECMO depression (versus control group)	1.52 (1.17, 1.96)	.002
Underlying anxiety disorder	1.15 (0.95, 1.39)	.140
Underlying PTSD	0.79 (0.46, 1.35)	.382
Age, years	1.04 (1.04, 1.05)	<.001
Sex, male	1.19 (0.99, 1.43)	.064
Residence at ECMO therapy		
Capital city (Seoul)	1	
Other metropolitan city	0.86 (0.66, 1.12)	.267
Other area	0.97 (0.79, 1.19)	.751
Year of ECMO therapy		
2006	1	
2007	0.69 (0.41, 1.16)	.165
2008	0.50 (0.29, 0.86)	.012
2009	0.54 (0.2, 0.89)	.017
2010	0.78 (0.49, 1.24)	.299
2011	0.74 (0.47, 1.15)	.186
2012	0.73 (0.47, 1.12)	.150
2013	0.53 (0.34, 0.81)	.004
2014	0.58 (0.387, 0.88)	.011
Annual income level at ECMO therapy		
Q1 (Lowest)	1	
Q2	0.94 (0.73, 1.22)	.652
Q3	0.66 (0.51, 0.84)	.001
Q4 (Highest)	0.86 (0.69, 1.07)	.185
Annual Case volume of ECMO therapy		
Q1 <14	1	
Q2: 15–29	0.94 (0.73, 1.22)	.652
Q3: 30–64	0.66 (0.51, 0.84)	.001
Q4 >64	0.86 (0.69, 1.07)	.185
Charlson comorbidity index, points	1.06 (1.02, 1.09)	.002
Underlying disability		
Mild to moderate	0.90 (0.70, 1.15)	.390
Severe	1.94 (1.55, 2.43)	<.001
Length of hospital stay, days	1.00 (0.99, 1.00)	.686
Duration of ECMO therapy, days	1.03 (1.02, 1.09)	.002
Main diagnosis at ECMO therapy		
Cardiovascular disease or Shock	1	
ARDS or respiratory failure	1.48 (1.07, 2.05)	.018
Post‐cardiac arrest	1.99 (1.33, 2.97)	.001
Others	2.33 (1.89, 2.87)	<.001

Note

Others: cancer, liver failure, sepsis, trauma, injury and burn, post‐procedures, connective tissue disease, GI tract disease, cerebrovascular disease, urinary tract disease, nervous system disease, endocrine disease, musculoskeletal disease, event at pregnancy or childbirth, poisoning or drug intoxication, hematologic disorder, congenital malformation, unknown.

Abbreviations: ARDS, acute respiratory distress syndrome; CI, confidence interval; ECMO, Extracorporeal membrane oxygenation; GI, gastrointestinalHR, hazard ratio; PTSD, post‐traumatic disorder.

### 3.3 Sensitivity and subgroup analyses

Table 4 shows the results of sensitivity analyses according to pre‐ and post‐depression with or without other psychiatric illness. Survivors of ECMO with post‐ECMO depression and anxiety or PTSD had a 2.01‐fold higher 5 year all‐cause mortality (HR, 2.01; 95% CI, 1.42–2.83; *p* < .001) than those in the control group. The survival plot derived from the multivariable model also showed a similar trend as shown in Figure S2. Table 5 shows the results of subgroup analyses according to main diagnosis at ECMO therapy. In the cardiovascular disease or shock group, post‐ECMO depression was associated with 1.54‐fold higher 5 year all‐cause mortality compared to that in the control group (HR, 1.54; 95% CI, 1.06–2.25; *p* = .025). In the postcardiac arrest group, pre‐ECMO depression was associated with a 14.65‐fold higher 5 year all‐cause mortality compared to that in the control group (HR, 14.65; 95% CI, 1.43–150.47; *p* = .024).

**TABLE 4:** Sensitivity analyses according to pre‐ and post‐ECMO depression with or without other psychiatric illness

Variable	Multivariable model	*p‐*value
	HR (95% CI)	
Pre‐depression		
Control group	1	
Pre‐ECMO depression without anxiety or PTSD (*n* = 159, 5.2%)	0.82 (0.53, 1.27)	.378
Pre‐ECMO depression with anxiety or PTSD (*n* = 135, 3.8%)	0.71 (0.44, 1.15)	.168
Post‐depression		
Control group	1	
Post‐ECMO depression without anxiety or PTSD (*n* = 196, 6.4%)	1.17 (0.81, 1.69)	.413
Post‐ECMO depression with anxiety or PTSD (*n* = 135, 4.4%)	2.01 (1.42, 2.83)	<.001

Note

Control group: ECMO survivors who were not diagnosed with depression before or after ECMO therapy.

Abbreviations: CI, confidence interval; HR, hazard ratio; PTSD, post‐traumatic disorder.

**TABLE 5:** Multivariable Cox regression model for 5 year all‐cause mortality in subgroups according to main diagnosis at ECMO therapy

Variable	Multivariable model	*p*‐value
	HR (95% CI)	
Cardiovascular disease or Shock group		
Pre‐ECMO depression (versus control group)	0.72 (0.29, 1.76)	.470
Post‐ECMO depression (versus control group)	1.54 (1.06, 2.25)	.025
ARDS or respiratory failure group		
Pre‐ECMO depression (versus control group)	0.56 (0.17, 1.79)	.328
Post‐ECMO depression (versus control group)	1.66 (0.71, 3.88)	.245
Post‐cardiac arrest group		
Pre‐ECMO depression (versus control group)	14.65 (1.43, 150.47)	.024
Post‐ECMO depression (versus control group)	0.10 (0.01, 1.34)	.082
Others group		
Pre‐ECMO depression (versus control group)	0.85 (0.35, 1.76)	.493
Post‐ECMO depression (versus control group)	1.43 (0.92, 2.21)	.109

Note

Other group: cancer, liver failure, sepsis, trauma, injury and burn, post‐procedures, connective tissue disease, GI tract disease, cerebrovascular disease, urinary tract disease, nervous system disease, endocrine disease, musculoskeletal disease, event at pregnancy or childbirth, poisoning or drug intoxication, hematologic disorder, congenital malformation, unknown. Control group: ECMO survivors who were not diagnosed with depression before or after ECMO therapy.

Abbrivations: HR, hazard ratio; CI, confidence interval; ECMO, Extracorporeal membrane oxygenation; ARDS, acute respiratory distress syndrome; GI, gastrointestinal.

## 4 DISCUSSION

This population‐based cohort study using data from the Korean NHIS database showed that 9.0% of survivors of ECMO were already diagnosed with depression before ECMO therapy, and 10.8% were newly diagnosed with depression within 365 days after ECMO therapy. While post‐ECMO depression was associated with a 1.52‐fold higher 5 year all‐cause mortality, pre‐ECMO depression was not. Additionally, this association was more evident in the post‐ECMO depression group with an underlying anxiety disorder or PTSD in the sensitivity analyses as well as in the post‐ECMO depression group that received ECMO therapy for cardiovascular disease or shock (potential VA ECMO group). Moreover, an older age, prolonged length of hospital stay, and underlying PTSD and anxiety disorder were associated with a higher incidence of post‐ECMO depression in our cohort. This is the first study to report that post‐ECMO depression might be a predictive factor for poor long‐term prognosis among survivors of ECMO. Our results suggest that survivors of ECMO who acquired and were diagnosed with depression within 365 days of ECMO therapy may be a high‐risk group requiring targeted interventions.

The 5 year survival rate among survivors of ECMO was 81.8% in this study, and it was similar to the 5 year survival rate of 87% reported in a previous study that focused on the 5 year survival rate among survivors of ECMO with respiratory failure and sepsis (von Bahr et al., 2017). A recent study reported that the prevalence of depression had increased from 2.8% in 2002 to 5.3% in 2013 among the South Korean population (Kim et al., 2020), which is relatively lower than prevalence of pre‐ECMO depression (9.0%) in our study. Our study focused on the critically ill and survivors of ECMO. Thus, the prevalence of depression in the participants of our study might be higher than that of the general adult population in South Korea.

Our study seems to report a lower prevalence (10.8%) of post‐ECMO depression than previous studies (15%–42%) (O'Brien et al., 2020; Sanfilippo et al., 2019; Sylvestre et al., 2019). However, the differences in the study design between our study and previous studies should be considered while interpreting this difference in prevalence (O'Brien et al., 2020; Sanfilippo et al., 2019; Sylvestre et al., 2019). Our results were based on objective factors, such as registered ICD‐10 codes in the NHIS database, while previous studies reported prevalence based on the results of surveys. Both methods have limitations. Specifically, prevalence assessment might have suffered from selection bias due to nonresponse to the surveys (Rupp et al., 2002). Indeed, the proportions of survivors of ECMO who responded to the survey in these studies were 68.4% (13/19) (O'Brien et al., 2020), 76.7% (23/30) (Orbo et al., 2019), and 86.8% (33/38) (Sanfilippo et al., 2019). However, our study might have suffered from detection bias (Sjolander et al., 2008) because we used the registered ICD‐10 codes for depression. For example, survivors of ECMO living in areas with better hospital or outpatient clinic access would be more likely to have their depression diagnosed by a medical professional than those living in areas with poor access to medical facilities. Considering these biases, more research is needed in the future regarding the prevalence of depression among survivors of ECMO.

The relationship between post‐ECMO depression and poor long‐term survival is the most important finding of this study. Some assumptions might be applied to explain this phenomenon. Many complications have been reported among patients following ECMO therapy, with central nervous system (CNS) complications having an incidence of 8% (Zangrillo et al., 2013). These complications include ischemic and hemorrhagic brain injury that may result in increased mortality (Le Guennec et al., 2018). Ischemic damage is known to increase the development of depression in the poststroke period through a neuroinflammatory mechanism (Robinson & Spalletta, 2010). This systemic and neuroinflammation may lead to a breakdown of the blood‐brain barrier, which results in an increased susceptibility of the CNS to certain drugs, such as sedatives and opioids. Thus, depression can develop as a consequence of systemic inflammation and neurotransmitter imbalances (Battle et al., 2015). Inflammatory responses due to the widespread activation of the innate immune system by ECMO therapy have been reported (Millar et al., 2016). Therefore, post‐ECMO depression might reflect the sequelae of CNS complications among survivors of ECMO. Neurologic complications among critically ill patients were risk factors for higher long‐term mortality (Sonneville et al., 2011), Based on this, the relationship between CNS complications following ECMO, post‐ECMO depression, and long‐term mortality might be understood.

In addition, the impact of stress on the development of depression among survivors of ECMO should be considered. A previous study reported that two‐fifths of survivors of an admission to the ICU experienced unemployment of up to 12 months following discharge (Kamdar et al., 2020). Furthermore, 71% of American survivors of ARDS experienced lost earnings (Kamdar et al., 2017). Unemployment is known to be closely related to depression (Dooley et al., 1994). Thus, in addition to the physiologic sequelae of ECMO therapy, the development of post‐ECMO depression needs to be understood from a social perspective. Since depression is also a known risk factor for increased mortality in the general population (Gilman et al., 2017), the prevention and treatment of post‐ECMO depression will be important for survivors of ECMO.

Another finding in the sensitivity analysis also showed that the post‐ECMO depression group with other psychiatric illness (anxiety disorder and PTSD) had increased 5 year all‐cause mortality among survivors of ECMO. The co‐existence of other such psychiatric illnesses suggests that the ECMO patients had more severe depression than those without psychiatric illness (Zimmerman et al., 2018), and this might have influenced the increased 5 year all‐cause mortality among survivors of ECMO. Thus, the patients with post‐ECMO depression with other psychiatric illnesses, such as anxiety disorder and PTSD, might be at a higher risk for poorer long‐term survival.

This study has several limitations. First, certain important variables, including body mass index, alcohol consumption, and smoking history, were not included in the analysis because the necessary information was not available in the NHIS database. Second, we defined comorbidities using their ICD‐10 codes to calculate the CCI; however, the ICD‐10 codes might not accurately reflect the actual diseases in the study population. Third, we did not distinguish between VA and VV ECMO in this study due to the limited prescription codes for ECMO in South Korea. Therefore, information on the difference in the prevalence of depression between VA and VV ECMO is missing from our study. Fourth, as mentioned above, our analysis of the prevalence of depression might have suffered from detection bias due to its use of ICD‐10 codes in the NHIS database. Lastly, we did not assess the cause of 5 year mortality among survivors of ECMO due to the limitations of our data source.

In conclusion, our study showed that 9.0% of survivors of ECMO were already diagnosed with depression before undergoing ECMO therapy, while 10.8% were newly diagnosed with depression within 365 days after ECMO therapy. Post‐ECMO depression was associated with an increased 5 year all‐cause mortality, but pre‐ECMO depression was not. Our results suggest that survivors of ECMO who acquired and were diagnosed with depression within 365 days of ECMO therapy may be a high‐risk group. Future studies are needed to investigate strategies to decrease post‐ECMO depression to improve both quality of life and long‐term survival among survivors of ECMO.

## ACKNOWLEDEGMENT

None.

## CONFLICTS OF INTEREST

None declared.

### PEER REVIEW

The peer review history for this article is available at https://publons.com/publon/10.1002/brb3.2218.

## Supporting information

Fig S1

Fig S2

Table S1

## Data Availability

The data that support the findings of this study are available on request from the corresponding author. The data are not publicly available due to privacy or ethical restrictions.
