# Supplementary figures and images for: Long‐term mortality associated with depression among South Korean survivors of extracorporeal membrane oxygenation
Source: Brain Behav. 2021 May 30;11(7):e02218. doi: 10.1002/brb3.2218 (PMC8323046; doi:10.1002/brb3.2218)

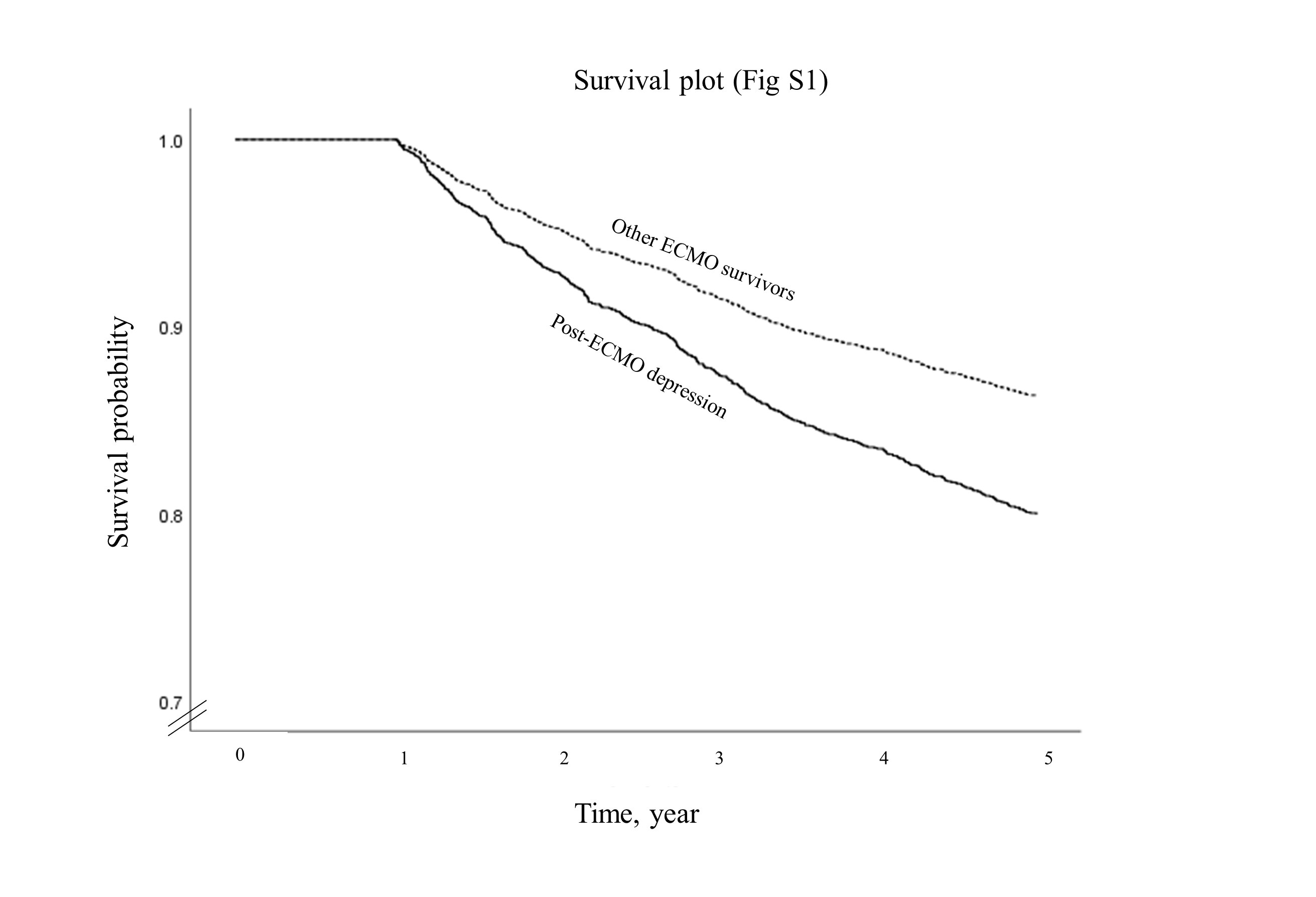

Supplement: Supplementary file 1 — Fig S1 [file BRB3-11-e02218-s002.tif]

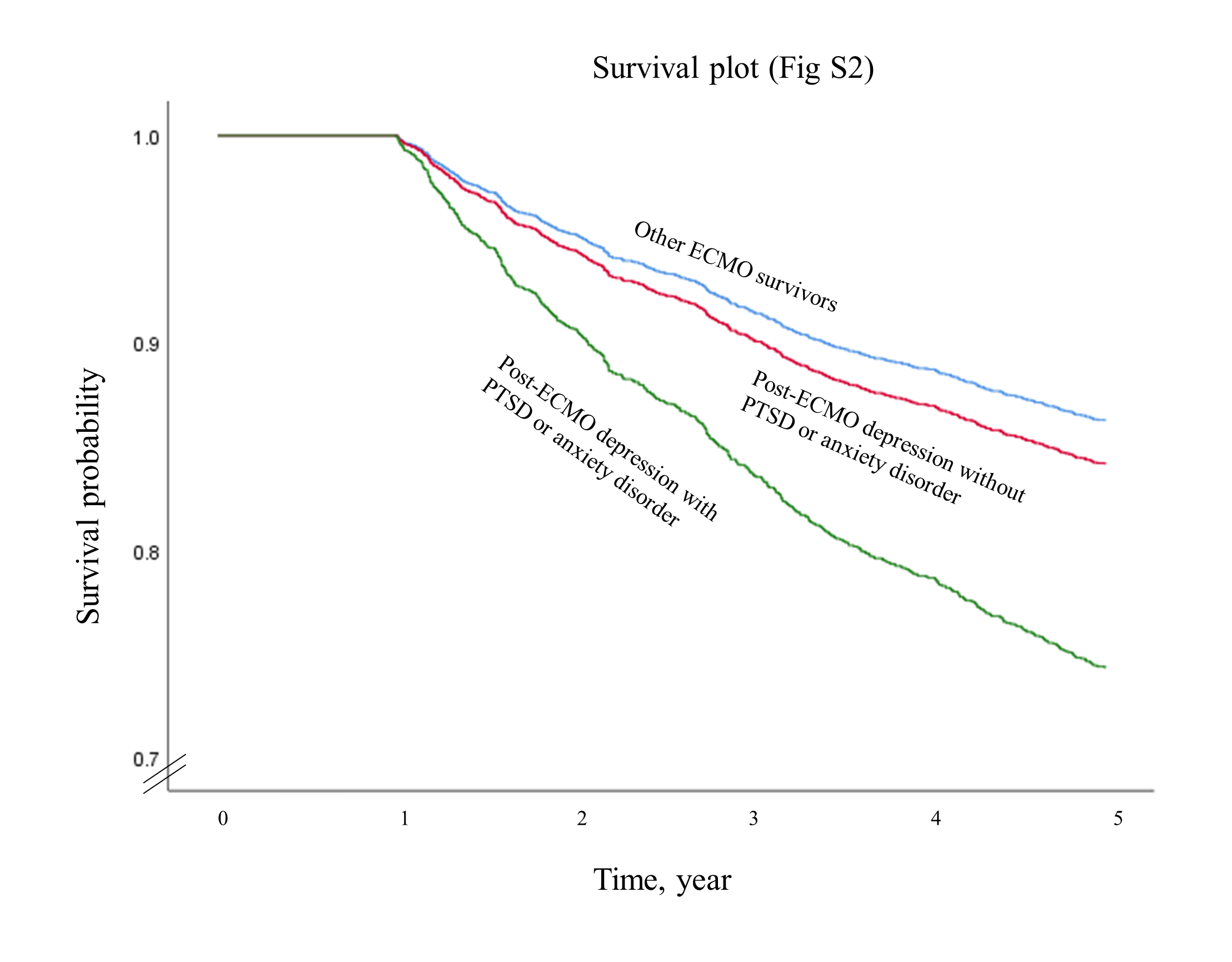

Supplement: Supplementary file 2 — Fig S2 [file BRB3-11-e02218-s001.tif]
